# Appropriate cardiopulmonary resuscitation duration and predictors of return of spontaneous circulation in traumatic cardiac arrest

**DOI:** 10.1186/s12873-025-01219-7

**Published:** 2025-04-14

**Authors:** Dongmin Seo, Inhae Heo, Kyoungwon Jung, Hohyung Jung

**Affiliations:** 1 Division of Trauma Surgery, Department of Surgery, Ajou University School of Medicine, 164 Worldcup-ro, Yeongtong-gu, Suwon-si, Gyeonggi-do 16499 Republic of Korea; 2 Regional Trauma Center of Southern Gyeong-gi Province, Ajou University School of Medicine, Suwon, Republic of Korea

**Keywords:** Wound and injuries, Heart arrest, Cardiopulmonary resuscitation, Return of spontaneous circulation

## Abstract

**Background:**

Despite advances in trauma care, traumatic cardiac arrest (TCA) shows significantly poorer outcomes compared to non-traumatic cardiac arrest, with mortality rates exceeding 96%. However, no standardized protocol exists for appropriate cardiopulmonary resuscitation (CPR) duration in TCA. This study aimed to establish evidence-based CPR duration thresholds and identify factors associated with return of spontaneous circulation (ROSC) in TCA patients.

**Methods:**

We conducted a retrospective observational study using a single-centre trauma registry of adult patients with TCA between January 2021 and December 2023. Univariate analysis was used to identify differences in the baseline and outcome variables between the ROSC and no-ROSC groups. We performed multivariable logistic regression analysis to identify factors independently associated with ROSC. We also investigated the appropriate cutoff time of pre-hospital and total CPR duration for ROSC (the CPR duration that has maximum sensitivity and specificity for ROSC).

**Results:**

In total, 422 patients with TCA were included, of whom 250 were eligible for analysis. The proportion of patients with ROSC was 22.4% (*n* = 56), and trauma bay/emergency department mortality and in-hospital mortality rates were 80.8% (*n* = 202) and 97.2% (*n* = 243), respectively. Factors associated with ROSC included alert mental status in the field, as indicated by verbal response (adjusted odds ratio [OR], 0.07; 95% confidence interval [CI], 0.01–1.12; *p* = 0.06), pain response (OR, 0.03; 95% CI, 0.01–0.43; *p* = 0.009), and unresponsiveness (OR, 0.04; 95% CI, 0.01–0.44; *p* = 0.009) and non-asystolic initial rhythms, such as pulseless electrical activity (OR, 4.26; 95% CI, 1.92–9.46; *p* < 0.001), shockable rhythm (OR, 14.26; 95% CI, 1.44–141.54; *p* = 0.023), pre-hospital CPR duration (OR, 0.90; 95% CI, 0.85–0.95), and total CPR duration (OR, 0.88; 95% CI, 0.84–0.92; *p* < 0.001). The upper limits of pre-hospital and total CPR durations for achieving a probability of ROSC < 1% were 23 and 30 min, respectively, whereas those for a cumulative portion of ROSC > 99% were 27 and 38 min, respectively. Among the survivors (*n* = 7), six had favourable functional outcomes at discharge.

**Conclusions:**

This study provides evidence-based CPR duration thresholds in TCA, demonstrating that resuscitation efforts beyond 27 min in prehospital settings and 38 min in total were futile. Additionally, an alert mental status in the field and non-asystolic initial rhythm were identified as positive predictors of ROSC. These findings may help guide appropriate duration of resuscitation efforts in TCA.

## Background

Traumatic cardiac arrest (TCA) is a life-threatening condition that poses substantial challenges in terms of management and outcomes. Despite advances in trauma care, survival rates for TCA remain alarmingly low. A 2012 systematic review of 47 studies reported an overall mortality rate of 96.7% [1]. This finding has been echoed in a more recent review, indicating an overall mortality rate of 96.2% [2]. Given the exceedingly low survival rates and substantial implications of TCA, there has been considerable debate regarding the initiation, continuation, and termination of cardiopulmonary resuscitation (CPR). In one report, resuscitative efforts were terminated if there were no signs of death [3]. In another report, trauma victims with asystole or agonal electrical cardiac activity were pronounced dead [4]. These recommendations underscore the necessity to balance costs, risks, and benefits in TCA management [5], with a lack of clear consensus on when to cease efforts.

The European Resuscitation Council (ERC) has developed an algorithm for TCA management that emphasises addressing reversible causes and performing resuscitative thoracotomy (RT) within 15 min of losing vital signs [6]; however, evidence of survivors beyond these guidelines exists [7], complicating the establishment of clear protocols for TCA management. Furthermore, although most trauma centres implement individualised protocols for determining the duration of CPR in patients with TCA, these protocols are not standardised and lack robust supporting evidence. Among the crucial factors in TCA, CPR duration has emerged as a pivotal determinant of outcomes, yet research specifically addressing this aspect remains limited.

While CPR duration is recognized as a critical determinant of outcomes in trauma care, there is no standardized protocol for appropriate resuscitation duration in TCA. This study aimed to establish evidence-based CPR duration thresholds and identify factors associated with ROSC in TCA patients, potentially providing objective criteria for termination of futile resuscitation efforts.

## Methods

### Study design and setting

This single-centre retrospective observational study was conducted using data from the Korean Trauma Data Bank (KTDB) registry and medical records from Ajou Trauma Registry between January 2021 and December 2023. Patients who underwent TCA were included in this study. TCA occurs when the heart stops owing to blunt or penetrating trauma. Drowning, hanging, and burning cases were excluded from this study. Patients aged < 16 years who were transferred from another hospital, dead on arrival (DOA) or for whom medical records were missing were excluded. We collected data on baseline characteristics, mechanism of injury, witnessed arrest, first documented rhythm, first documented mental status, interventions during CPR, CPR duration, and outcomes. Pre-hospital CPR duration and achieving of ROSC were verified through review of Emergency Medical Services (EMS) records, which are included in both the Korean Trauma Data Bank (KTDB) and Ajou Trauma Registry.

The study protocol was approved by the institutional review board of Ajou University Hospital on January 30, 2024 (No. AJOUIRB-DB-2024-067), and data were accessed for research purposes from this date onward. The institutional review board waived the requirement for informed consent due to the observational nature of the research. Additionally, the patients’ information was anonymised and de-identified prior to analysis.

### Exposure and outcomes

The primary exposure was the CPR duration, divided into pre-hospital, in-hospital, and total CPR durations. Pre-hospital CPR duration was defined as the time from CPR initiation by emergency medical services to achieving pre-hospital ROSC or hospital arrival. In-hospital CPR duration was defined as the time from CPR initiation at the hospital (trauma bay or emergency department [ED]) to the achieving ROSC or discontinuation of resuscitation. The total CPR duration was defined as the time from CPR initiation to the time of achieving ROSC or discontinuation of resuscitation.

The primary outcome was ROSC, which was defined as a clinical assessment indicating signs of life, such as a palpable pulse or generation of blood pressure regardless of its duration [8]. Secondary outcomes included trauma bay or ED mortality and overall mortality.

### Statistical analysis

Univariate analysis was used to identify differences in the baseline and outcome variables between patients who achieved ROSC and those who did not. Categorical variables were compared using the Chi-squared test or Fisher’s exact test. The Mann–Whitney U test was used to compare medians for continuous variables. Results are reported as numbers and percentages for categorical variables or medians with interquartile ranges (IQR) for continuous variables. Clinically relevant parameters, all with *p* < 0.1 upon univariate analysis, were included in the regression model. Univariate and multivariate logistic regression analyses were conducted to identify independent predictors of ROSC. Results are reported as odds ratios (ORs) with 95% confidence intervals (CIs). Correlations between the variables were tested using multicollinearity analysis. The area under the receiver operating characteristic curve with 95% CI was used to assess the accuracy of the test.

The dynamic probability and cumulative proportion of ROSC were calculated for all eligible participants and stratified by significant variables. A dynamic probability of ROSC < 1% indicated the proportion of ROSC < 1%. A cumulative proportion > 99% refers to 99% of patients with ROSC. We measured the appropriate cutoff time for pre-hospital and total CPR duration, which is the CPR duration with maximum sensitivity and specificity for ROSC. The shortest distance between each point on the receiver operating characteristic curve and the upper left corner was considered the appropriate cutoff CPR duration for ROSC.

Analyses were performed using R software version 4.3.0 (The R Foundation for Statistical Computing, Vienna, Austria) and SPSS version 25.0 (SPSS Inc., Chicago, IL, USA). Statistical significance was set at *p* < 0.05.

## Results

**Fig. 1:**
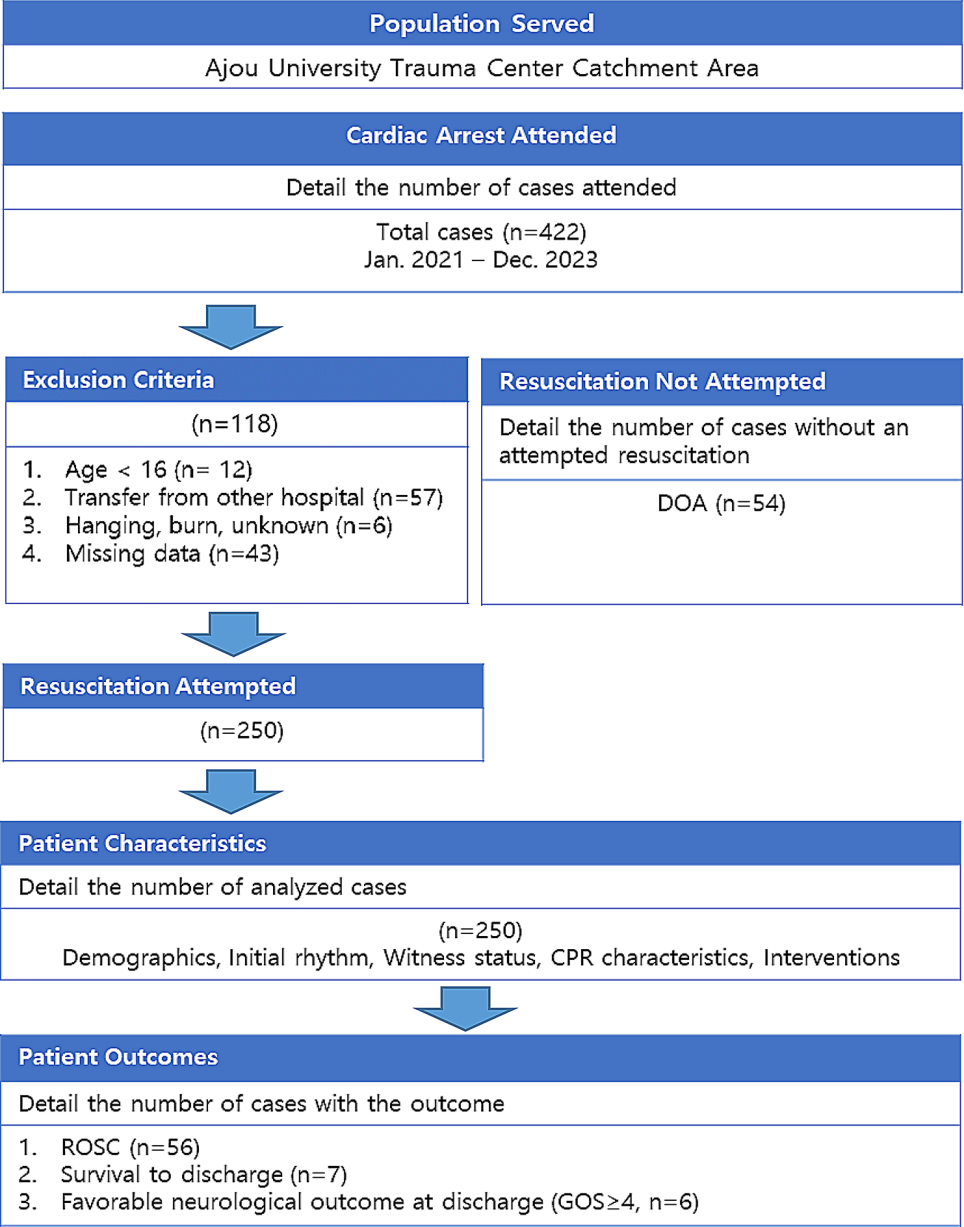
Flow of study. DOA, dead on arrival; CPR, cardiopulmonary resuscitation; ROSC, return of spontaneous circulation; GOS, Glasgow outcome scale

During the study period, 422 patients with TCA were included in the registry. After applying the exclusion criteria, 250 patients were eligible for analysis (Fig. 1). The baseline characteristics, pre-hospital, and in-hospital information of the study population are presented in Table 1. The median age was 60 years (30–65 years), and 65.6% (*n* = 164) of the patients were male. Blunt trauma accounted for the majority of cases (97.2%, *n* = 243), with falls being the most common mechanism of injury (61.6%, *n* = 154), followed by road traffic accidents (RTAs) (32.8%, *n* = 82). Witnessed arrests occurred in 28% (*n* = 70) of the cases. The overall mortality rate was 97.2% (*n* = 243), whereas the trauma bay or ED mortality rate was 80.8% (*n* = 202). The median pre-hospital CPR duration was 15 min (IQR, 10–21), and the total CPR duration was 24 min (IQR, 18–29). Table 1 also illustrates the interventions performed during CPR and emergency procedures. Bleeding was the most commonly presumed cause of arrest (33.6%, *n* = 84), followed by brain injury (26.8%, *n* = 67) and unknown causes (20.8%, *n* = 52).

**Table 1:** Baseline characteristics and outcomes of all patients

Variables	Each proportion of patients(%) or median (IQR)
**Total**	250
**Age**,** year**	60 (30–65)
> 65	63 (25.2)
**Sex**,** male**	164 (65.6)
**Type of Injury**	
Blunt	243 (97.2)
Penetrating	7 (2.8)
**Mechanism of Injury**	
RTA	82 (32.8)
Fall	154 (61.6)
Stab wound	7 (2.8)
Other	7 (2.8)
**Transport**	
Helicopter	11 (4.4)
Ground ambulance	239 (95.6)
**Field initial AVPU**	
A	13 (5.2)
V	10 (4.0)
P	15 (6.0)
U	212 (84.8)
**Witnessed arrest**	70 (28.0)
**First recorded rhythm**	
Asystole	143 (57.2)
PEA	101 (40.4)
VF/VT	6 (2.4)
**CPR duration**,** min**	
Prehospital CPR duration	15 (10–21)
In-hospital CPR duration	6 (4–10)
Total CPR duration	24 (18–29)
**Interventions during CPR**	
Airway management (SGA insertion or intubation)	246 (98.4)
Vascular access	239 (95.6)
IV	126 (50.4)
IO	113 (45.2)
Finger/Needle thoracostomy	42 (16.8)
Resuscitative thoracotomy	73 (29.2)
**Emergency interventions**	
REBOA	8 (3.2)
Massive transfusion	32 (12.8)
Emergency operation	11 (4.4)
Angioembolization	3 (1.2)
**ROSC**	56 (22.4)
**Time to Death (min)**	285 (140–1634)
**T-bay/ED Mortality**	202 (80.8)
**In-hospital Mortality**	243 (97.2)
**Presumed cause of arrest**	
Airway problem	17 (6.8)
Bleeding	84 (33.6)
Chest injury	30 (12.0)
Brain injury	67 (26.8)
Unknown	52 (20.8)

Continuous variables are presented as median (interquartile range). Categorical variables are presented as numbers (percentages)

RTA, Road traffic accident; A, Alert; V, Verbal response; P, Pain response; U, Unresponsive; PEA, Pulseless electrical activity; VF, Ventricular fibrillation; VT, Ventricular tachycardia; CPR, Cardiopulmonary resuscitation; ROSC, Return of spontaneous circulation; SGA, Supraglottic airway device ((i-gel™, Intersurgical Ltd., Wokingham, UK); IV, Intravenous; IO, Intraosseous; REBOA, Resuscitative endovascular balloon occlusion; ED, Emergency department

ROSC was achieved in 22.4% (*n* = 56) of the patients. Table 2 compares the characteristics and outcomes between the ROSC and no-ROSC groups. Patients who achieved ROSC had a higher proportion of males (75% vs. 62.9%, *p* = 0.02), RTAs (55.3% vs. 26.3%, *p* < 0.001), alert mental status in the field (19.6% vs. 1%, *p* < 0.001), witnessed arrests (57.1% vs. 19.6%, *p* < 0.001), and pulseless electrical activity (PEA) as the initial rhythm (71.4% vs. 31.4%, *p* < 0.001). The ROSC group also had significantly shorter pre-hospital CPR duration (median, 7.5 vs. 16 min, *p* < 0.001) and total CPR duration (median, 15 vs. 25 min, *p* < 0.001) than the no-ROSC group.

**Table 2:** Comparison of characteristics and outcomes between ROSC and no-ROSC patients

Variables	Total (*n* = 250)	ROSC (*n* = 56)	No-ROSC (*n* = 194)	*p*-value
**Age**,** year**	60 (30–65)	58.5 (37.5–66)	49 (28–64)	0.109
> 65	63 (25.2)	16 (28.6)	47 (24.2)	0.458
**Sex**,** male**	164 (65.6)	42 (75.0)	122 (62.9)	0.020
**Type of Injury**				0.691
Blunt	243 (97.2)	51 (91.1)	192 (99.0)	
Penetrating	7 (2.8)	5 (8.9)	2 (1.0)	
**Mechanism of Injury**				
RTA	82 (32.8)	31 (55.3)	51 (26.3)	< 0.001
Fall	154 (61.6)	20 (35.7)	134 (69.1)	< 0.001
Stab wound	7 (2.8)	2 (3.6)	5 (2.6)	0.691
Other	7 (2.8)	3 (5.4)	4 (2.1)	0.188
**Transport**				0.256
Helicopter	11 (4.4)	7 (12.5)	4 (2.1)	
Ground ambulance	239 (95.6)	49 (87.5)	190 (97.9)	
**Field initial AVPU**				
A	13 (5.2)	11 (19.6)	2 (1.0)	< 0.001
V	10 (4.0)	4 (7.1)	6 (3.1)	0.173
P	15 (6.0)	5 (8.9)	10 (5.2)	0.295
U	212 (84.8)	36 (64.3)	176 (90.7)	< 0.001
**Witnessed arrest**	70 (28.0)	32 (57.1)	38 (19.6)	< 0.001
**First recorded rhythm**				
Asystole	143 (57.2)	13 (23.2)	130 (67.0)	< 0.001
PEA	101 (40.4)	40 (71.4)	61 (31.4)	< 0.001
VF/VT	6 (2.4)	3 (5.4)	3 (1.5)	0.101
**CPR duration**,** min**				
Prehospital CPR duration	15 (10–21)	7.5 (0–16.5)	16 (12–22)	< 0.001
In-hospital CPR duration	6 (4–10)	5 (2.75–8)	6 (4.25–10)	0.007
Total CPR duration	24 (18–29)	15 (5.75–24)	25 (20–30)	< 0.001

Continuous variables are presented as median (interquartile range). Categorical variables are presented as numbers (percentages)

RTA, Road traffic accident; A, Alert; V, Verbal response; P, Pain response; U, Unresponsive; PEA, Pulseless electrical activity; VF, Ventricular fibrillation; VT, Ventricular tachycardia; CPR, Cardiopulmonary resuscitation; ROSC, Return of spontaneous circulation

The results of the multivariate logistic regression analysis for factors associated with ROSC are shown in Table 3. Alert mental status in the field, as indicated by verbal response (OR, 0.07; 95% CI, 0.01–1.12; *p* = 0.06), pain response (OR, 0.03; 95% CI, 0.01–0.43; *p* = 0.009), and unresponsiveness (OR, 0.04; 95% CI, 0.01–0.44; *p* = 0.009), as well as non-asystolic initial rhythms such as PEA (OR, 4.26; 95% CI, 1.92–9.46; *p* < 0.001) and shockable rhythm (OR, 14.26; 95% CI, 1.44–141.54; *p* = 0.023), were significantly associated with ROSC. Additionally, both pre-hospital CPR duration (OR, 0.90; 95% CI, 0.85–0.95; *p* < 0.001) and total CPR duration (OR, 0.88; 95% CI, 0.84–0.92; *p* < 0.001) were inversely associated with the likelihood of achieving ROSC.

**Table 3:** Multivariable predictive models for ROSC

	Prognostic variables	OR	95%CI	*p*-value
ROSC	**Field initial AVPU**			
	A	1(reference)		
	V	0.07	0.01–1.12	0.060
	P	0.03	0.01–0.43	0.009
	U	0.04	0.01–0.44	0.009
	**First recorded rhythm**			
	Asystole	1(reference)		
	PEA	4.26	1.92–9.46	< 0.001
	VF/VT	14.26	1.44–141.54	0.023
	**Prehospital CPR duration**	0.90	0.85–0.95	< 0.001
	**In-hospital CPR duration**	0.82	0.75– 0.90	< 0.001
	**Total CPR duration**	0.88	0.84–0.92	< 0.001

OR, Odds ratio; CI, Confidence intervals; CPR, Cardiopulmonary resuscitation; ROSC, Return of spontaneous circulation; PEA, Pulseless electrical activity

R2: Cox and Snell R2 = 0.451, Hosmer–Lemeshow goodness-of-fit = 0.137

**Fig. 2:**
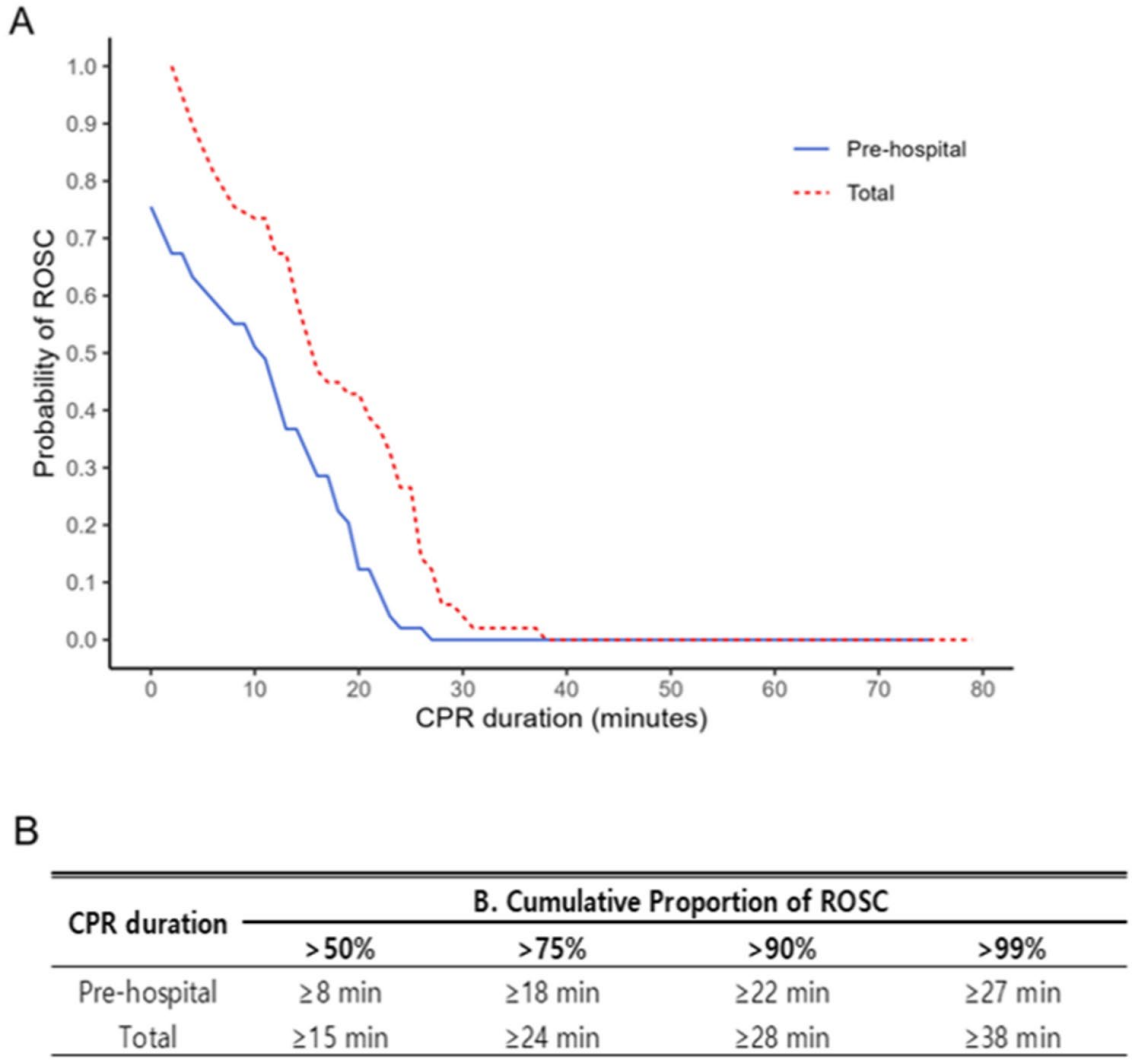
Dynamic probability (**a**) and cumulative proportion (**b**) of ROSC for prehospital and total CPR duration. CPR, cardiopulmonary resuscitation; ROSC, return of spontaneous circulation

The dynamic probability and cumulative proportion of ROSC according to the pre-hospital and total CPR durations are presented in Fig. 2. The upper limits of pre-hospital and total CPR durations for achieving a probability of ROSC < 1% were 23 and 30 min, respectively, whereas those for a cumulative proportion of ROSC > 99% were 27 and 38 min, respectively.

Among the study population, seven patients (2.8%) survived to discharge. The detailed characteristics of survivors are presented in Table 4. Of these, 85.7% (*n* = 6) were male, with a predominant age range of 60–69 years. All survivors demonstrated significant thoracic trauma (Abbreviated Injury Scale score ≥ 3). The mechanism of arrest was attributed to exsanguination in three cases and thoracic pathology in four cases. Survivors demonstrated significantly shorter CPR durations (median 4 min, IQR 2–16) compared to non-survivors. Despite various in-hospital complications including acute kidney injury, hospital-acquired pneumonia, and in-hospital cardiac arrest, 85.7% (*n* = 6) of survivors achieved favorable neurological outcomes (Glasgow Outcome Scale 4–5) at discharge.

**Table 4:** Characteristics and outcomes of survivors of traumatic cardiac arrest

#	Age range	Sex	Type of injury	Mechanism of injury	AVPU	Rhythm	Witnessed	CPR time (pre/in/total)
1	60–69	M	RTA	Blunt	Alert	PEA	Y	4/22/26
2	60–69	M	Fall	Blunt	Unresponsive	VF/VT	Y	4/0/4
3	40–49	M	Penetrating	Penetrating	Alert	PEA	N	0/8/8
4	60–69	F	Fall	Blunt	Alert	PEA	N	0/2/2
5	70–79	M	Fall	Blunt	Unresponsive	PEA	Y	2/0/2
6	20–29	M	Fall	Blunt	Unresponsive	VF/VT	Y	16/0/16
7	20–29	M	Penetrating	Penetrating	Unresponsive	PEA	N	1/1/2

#	AIS	Presumed cause of arrest	ISS	RTS	TRISS	Interventions during CPR	Emergency interventions
1	Chest AIS: 3 Abdomen&Pelvis AIS: 3 Pelvis&extremities AIS: 5 External AIS: 1	Bleeding	43	3	0.032587	SGA IV	Massive transfusion EDT Emergency operation
2	Chest AIS: 3 Abdomen&Pelvis AIS: 2 Pelvis&extremities AIS: 2 External AIS: 1	Chest injury	17	12	0.949841	O2 mask IV	-
3	Chest AIS: 4	Chest injury	16	3	0.876522	Intubation IV	Massive transfusion EDT
4	Head AIS: 3 Chest AIS: 4 Abdomen&Pelvis AIS: 2 Pelvis&extremities AIS: 5 External AIS: 1	Bleeding	50	10	0.369491	Cricothyrotomy IV	Massive transfusion EDT Emergency operation
5	Head AIS: 3 Chest AIS: 4 Abdomen&Pelvis AIS: 2 Pelvis&extremities AIS: 3 External AIS: 1	Chest injury	34	8	0.282276	O2 mask	Massive transfusion
6	Head AIS: 1 Chest AIS: 3 External AIS:1	Chest injury	11	8	0.925318	O2 mask	Intubation
7	Chest AIS: 4 Abdomen&Pelvis AIS: 4	Bleeding	32	12	0.934594	Intubation IV	Massive transfusion Emergency operation

#	MV days	ICU LOS	Hospital LOS	Hospital event	GOS
1	53	53	53	AKI, HAP, Sepsis	3
2	0	4	19	Unplanned return to the ICU	5
3	1	4	38	IHCA	4
4	13	13	41	ARDS, IHCA, CLABSI	4
5	13	22	41	Unplanned intubation, HAP	4
6	17	20	26	Unplanned intubation, HAP	5
7	2	5	65	Unplanned return to the OR, unplanned return to the ICU	5

AVPU, Alert, Verbal, Pain, Unresponsive; CPR, Cardiopulmonary resuscitation; RTA, Road traffic accident; PEA, Pulseless electrical activity; VF, Ventricular fibrillation; VT, Ventricular tachycardia

AIS, Abbreviated injury scale; ISS, Injury severity score; RTS, Revised trauma score; TRISS, Trauma score and injury severity score; CPR, Cardiopulmonary resuscitation; EDT, Emergency department thoracotomy; IV, Intravenous; SGA, Supraglottic airway device ((i-gel™, Intersurgical Ltd., Wokingham, UK)

MV, Mechanical ventilation; ICU, Intensive care unit; LOS, Length of stay; GOS, Glasgow outcome scale; AKI, Acute kidney injury; HAP, Hospital acquired pneumonia; IHCA, In-hospital cardiac arrest; CLABIS, Central line-associated bloodstream infection; OR, Operation room

## Discussion

In this single-center retrospective observational study, we demonstrated that CPR duration significantly influences outcomes in TCA patients. Furthermore, we identified that non-asystolic initial rhythms and alert mental status in the field were significant predictors of ROSC. Most importantly, our analysis established evidence-based temporal thresholds for resuscitation efforts in TCA, providing objective criteria for termination of futile resuscitation attempts.

TCA incidents are less common than cardiac-related emergencies but are associated with significantly lower survival rates at hospital admission (14.2% for TCA vs. 29.3% for medical cardiac arrest, *p* < 0.001). This disparity can be attributed to several factors, including less frequent eyewitness accounts and the prevalence of non-shockable rhythms in TCA [9, 10]. Consequently, management of TCA requires a distinct approach that focuses on addressing reversible causes, as emphasised in the ERC guidelines [6]. This may involve interventions such as resuscitative endovascular balloon occlusion of the aorta (REBOA) or RT. However, there is a paucity of research specifically addressing TCA, highlighting the need for further investigation.

In our study, blunt injuries accounted for the majority of cases (97.2%), which is higher than the rates reported in other studies, ranging from 56.6 to 83.5% [11–13]. This finding can be attributed to the fact that blunt injuries are the predominant type of trauma in South Korea [14, 15], where gun possession is illegal, and most injuries result from falls and RTAs. Although univariate analysis revealed significant differences in sex, mechanism of injury, and witnessed arrest, these factors did not retain significance in the multivariate analysis. The overall mortality rate was 97.2%, with 80.8% of deaths occurring in the trauma bay or ED, consistent with the findings of a recent systematic review [2].

Previous studies have identified predictors of ROSC in patients with TCA. A recent study found that non-asystole rhythm upon mobile medical team arrival is associated with higher ROSC rates [16]. Similarly, a meta-analysis reported that cardiac motion on ultrasound or a shockable initial rhythm is a predictor of ROSC [17]. Another study highlighted that advanced life support by physicians is associated with increased survival in patients with TCA [18]. Our results corroborate these findings, emphasising the significance of initial rhythm as a predictor of ROSC.

Several studies have evaluated CPR duration and outcomes in non-traumatic cardiac arrests. Park et al. [19] established CPR duration thresholds associated with neurological outcomes in out-of-hospital cardiac arrest. Matsuyama et al. [20] conducted a nationwide multicenter investigation demonstrating that prolonged resuscitation yielded favorable neurological outcomes in selected non-traumatic cardiac arrest cases with specific clinical features. Additionally, Okubo et al. [21] analyzed the relationship between CPR duration and survival in in-hospital cardiac arrest. While these investigations provide valuable evidence for medical cardiac arrest resuscitation protocols, their findings have limited applicability to TCA due to distinct pathophysiological mechanisms. Despite the clinical significance of CPR duration in trauma care, evidence specific to TCA remains insufficient. Although one study reported survival rates of 4.3–36.4% in trauma patients receiving immediate CPR after arrival [22], validated temporal thresholds for TCA resuscitation have not been established. Our analysis addresses this evidence gap by defining specific CPR duration parameters for TCA, providing objective criteria for termination of resuscitation efforts in this unique patient population.

This study has several limitations. First, its retrospective and observational nature inherently involves unmeasured confounding factors. These include patient-specific factors (comorbidities, physiologic reserve, pre-injury medications, particularly anticoagulants), resuscitation-specific variables (compression quality, adherence to advanced life support protocols, provider experience), and system-based factors (transport time, resource availability). Second, the single-centre design and sample size limit the generalizability of our findings. Finally, the predominance of blunt trauma mechanisms in our cohort may not be applicable to populations with different injury patterns.

To validate these CPR duration benchmarks and outcome predictors across diverse TCA populations, larger, multi-institutional, prospective studies are warranted. Furthermore, exploring the impact of advanced resuscitative techniques, such as REBOA and RT, could help refine management protocols.

## Conclusions

In summary, this study provides evidence-based CPR duration thresholds in TCA, demonstrating that resuscitation efforts beyond 27 min in prehospital settings and 38 min in total were futile. Additionally, an alert mental status and non-asystolic initial rhythm were identified as positive predictors of ROSC. These findings establish objective parameters for resuscitation efforts in TCA patients, contributing to evidence-based protocols for the termination of futile resuscitation. Further prospective multi-center studies are warranted to validate these thresholds across diverse trauma populations.

## Data Availability

The datasets used and/or analyzed during the current study are available from the corresponding author on reasonable request.

## Abbreviations

TCA: Traumatic cardiac arrest
ROSC: Return of spontaneous circulation
CPR: Cardiopulmonary resuscitation
REBOA: Resuscitative endovascular balloon occlusion of the aorta
OR: Odds ratio
CI: Confidence interval
ERC: European Resuscitative Council
RT: Resuscitative thoracotomy
AVPU: Alert, Verbal, Pain, Unresponsive
CPR: Cardiopulmonary resuscitation
IV: Intravenous
EDT: Emergency department thoractomy
MV: Mechanical ventilation
ICU: Intensive care unit
LOS: Length of stay
AKI: Acute kidney injury
HAP: Hospital acquired pneumonia
IHCA: In-hospital cardiac arrest
ARDS: Acute respiratory distress syndrome
CLABSI: Central line-associated bloodstream infection
OR: Operation room
